# Canine leishmaniasis seroprevalence in asymptomatic dogs: results from an epidemiological study in endemic Mediterranean countries

**DOI:** 10.3389/fvets.2026.1821436

**Published:** 2026-05-11

**Authors:** Manuela Gizzarelli, Gaetano Oliva, Ines Balestrino, Alexandra Beck, Thomas Blondel, Alessia Crippa, Cécile Collignon, Valentina Foglia Manzillo

**Affiliations:** 1 Department of Veterinary Medicine and Animal Production, University “Federico II”, Naples, Italy; 2 Ceva Santé Animale, Libourne, France

**Keywords:** asymptomatic dog, canine leishmaniosis, epidemiology, Leishmania rapid test, Leishmania seroprevalence

## Abstract

**Introduction:**

Canine leishmaniosis (CanL), caused by *Leishmania infantum*, is a major zoonotic disease in Mediterranean countries, where dogs act as the primary reservoir for transmission by phlebotomine sand flies. A significant proportion of infected dogs remain in an asymptomatic subclinical (AS) state and may contribute to maintaining the parasite’s transmission cycle. Identifying these AS dogs is essential for disease surveillance and for implementing effective preventive strategies.

**Methods:**

From January to April 2024, the non-transmission period for sand flies, 548 clinically healthy dogs were enrolled in France, Greece, Italy, and Spain during routine veterinary visits. Dogs underwent clinical examination and rapid immunochromatographic testing for anti-*Leishmania* antibodies. Demographic and clinical variables were recorded, and statistical analyses were performed to explore associations between test positivity and factors including age, sex, body weight, sample matrix, and country of residence

**Results:**

Overall seroprevalence was 7.3%, with marked variation among countries: France (2.9%), Spain (3.2%), Italy (8.8%), and Greece (19%). Univariable analysis showed no significant association between rapid test positivity and age, sex, or body weight. In contrast, country and sample material (plasma vs serum) were significantly associated with test results. In the multivariable model, only the country remained a significant predictor. A relevant proportion of seropositive asymptomatic dogs was detected among owned healthy dogs in endemic European regions, highlighting their potential epidemiological role in sustaining *L. infantum* transmission.

**Discussion:**

These findings underscore the crucial role of veterinarians in promoting regular screening of clinically healthy dogs, increasing owner awareness of CanL, and encouraging the use of effective repellent-based parasiticides to reduce the risk of parasite transmission.

## Introduction

Leishmaniasis, caused by the protozoan *Leishmania infantum,* is a zoonotic infection with a broad distribution across both the Northern and Southern hemispheres, encompassing the Old and New Worlds. Among domestic species, infected dogs are considered the primary reservoir of *Leishmania infantum,* which is transmitted by phlebotomine sand fly vectors. However, this pathogen also infects a range of other domestic and wild animals. In the Mediterranean basin, leishmaniasis due to *Leishmania infantum* is one of the most prevalent vector-borne diseases in dogs (1). Canine leishmaniasis (CanL) is a progressive disease characterized by a broad spectrum of clinical presentations, ranging from a generally healthy status in asymptomatic subclinical (AS) infection to severe or even fatal disease. There is a lack of a standardized definition of the AS condition, which is often described as the absence of clinical signs on examination along with normal clinicopathological parameters. This stage of infection is typically characterized by the detection of parasite DNA by PCR and/or the appearance of low antibody titres. In these dogs, a strong cellular immune response persists, with balanced, tissue-specific immunity that does not cause immune-mediated tissue damage or subsequent disease. In *Leishmania-*endemic countries, the number of AS infected dogs is not known; however, they may represent more than 50% of infected animals (2). There is substantial evidence that AS infected dogs can be infectious to sand flies and may contribute to the maintenance of the *Leishmania* life cycle and to the transmission of the parasite to other mammals (3–9). For this reason, it is important to identify AS infected dogs within the whole canine population and to protect them with effective pyrethroid repellents against sand fly bites, thereby limiting the spread of the *Leishmania* parasite through this important animal reservoir. According to the WOAH *Terrestrial Animal Health Code*, the definition of asymptomatic *Leishmania*-infected dogs requires the direct demonstration of the parasite through cytological or histological identification, or the detection of its DNA using conventional or quantitative PCR methods. Nevertheless, in routine veterinary practice, the collection of invasive samples from asymptomatic dogs—such as bone marrow or lymph node aspirates, which are regarded as optimal for *Leishmania* DNA detection—is frequently limited by poor owner compliance. For this reason, the diagnosis of *Leishmania* infection is usually based on serology, which cannot be considered a definitive confirmation of infection status. The aim of the present study was to assess the seroprevalence of asymptomatic cases among healthy dogs living in endemic European countries, in order to reflect what typically occurs in daily practice.

## Materials and methods

Dogs were enrolled from January to April 2024, a period not typically associated with *Leishmania* transmission by sand flies, in four Mediterranean European countries (France, Greece, Italy, and Spain). Dogs were selected from those presented to private veterinary clinics for routine vaccination programs or periodic check-ups, in the absence of any clinical signs. Owners were asked to sign an informed consent form before any study procedures. A clinical examination was performed to rule out the presence of clinical signs. A clinical form was filled to record body weight (BW), age, sex, and the most recent parasiticide treatment administered. A rapid test based on an immunochromatographic technique (Uranotest®-*Leishmania*, URANO diagnostics, Spain, www.uranodiagnostics.com) was performed to assess the presence of antibodies against *Leishmania* spp. Moreover, 1 mL of blood was collected from the cephalic vein and immediately utilized to perform the test. Briefly, the procedure involves a test strip containing a circular well into which the sample is added. A purple test (T) line and a purple control (C) line appear after capillary migration of the sample in the case of a positive result. Only the C line appears when the sample is negative. The specificity and sensitivity of this test are 1 and 0.98, respectively, as demonstrated in a study that compared five different immunochromatographic tests (Villanueva-Saz et al., 2022). The practitioners informed the pet owners about the rapid test results and proposed to confirm the diagnosis with further analyses such as quantitative serology and/or specific PCR for *Leishmania* spp.

## Statistical analysis

Each variable was described both by country and overall. Quantitative variables were described using the number of observations, mean with standard deviation, median, interquartile range, and range. Qualitative variables were described using the number of observations and percentages. The distribution of some variables was compared between countries. For quantitative variables, the Kruskal–Wallis test was used to compare the distribution between countries, followed by pairwise Dunn’s test when the Kruskal–Wallis test was < 0.05. For qualitative variables with two categories, a logistic regression model was used with a log link (logit link for breed) and covariates were included as fixed effects. From each model, relative risks were estimated (odds ratios for breed), along with the corresponding 95% confidence interval and the associated *p*-value. *p*-values were adjusted using the Tukey method. A p-value of < 0.05 was considered statistically significant.

No statistical analyses were performed for parasiticides because essential information regarding treatment compliance and administration timing was not collected. The available data did not specify whether the product had been administered during the previous sand fly season or during the study period (January–March), which fell outside the sand fly season. Consequently, no robust conclusions could be drawn regarding the impact of the parasiticides used on test positivity.

## Results

**Figure 1:**
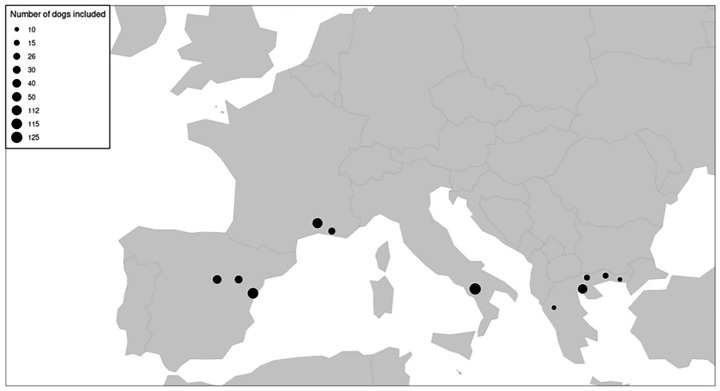
Location and number of enrolled dogs.

A total of 548 (*n* = 548) healthy dogs were recruited from France (*n* = 138), Greece (*n* = 100), Italy (*n* = 125), and Spain (*n* = 185) (Figure 1). The overall mean age was 5.3 ± 2.3 years [1.0; 10.2], and the mean BW was 19.8 ± 10.6 kg [2.0; 58.0]. The study population showed a relatively balanced sex distribution, with female individuals representing just over half of the animals (approximately 52%) (Table 1). The composition of the dog groups was homogeneous for all countries, except for BW. Dogs weighing more than 20 kg were significantly more prevalent in France (60.9%) than in Greece (34.0%). The last parasiticide administered was a repellent product against sand flies in 40.9% of animals overall, with proportions ranging from 30.0% in Greece to 49.0% in Spain. Non-repellent treatments (defined as treatments with no proven repellent/anti-feeding activity against sand flies) were the last ones administered in 30.8% of dogs, being commonly used in France and Italy but absent in Greece. Furthermore, 28.3% of animals received no treatment, with the highest proportion observed in Greece (70.0%) (Table 2). The material used for testing was whole blood (*n* = 17) or samples centrifuged to obtain plasma (*n* = 169) or serum (*n* = 362), with serum samples accounting for nearly 100% of the dogs tested in Greece and Italy (data not shown). A total of 40 dogs tested positive on the rapid test, corresponding to an overall seroprevalence of 7.3% (Table 3). Seroprevalence varied across countries, ranging from 2.9% in France and 3.2% in Spain to 8.8% in Italy and 19% in Greece.

**Table 1:** Characteristics of the dogs enrolled in the study.

Animal data		France *n* = 138	Greece *n* = 100	Italy *n* = 125	Spain *n* = 185	Total *n* = 548
Age (years)	Mean (SD)	5.1 (2.3)	5.1 (2.1)	5.3 (2.5)	5.5 (2.4)	5.3 (2.3)
	[Min; Max]	[1.4; 9.8]	[1.9; 9.7]	[1.0;10.2]	[2.0; 9.9]	[1.0;10.2]
Body weight (kg)	Mean (SD)	22.8 (10.5)	17.0 (5.7)	19.2 (9.5)	19.4 (12.8)	19.8 (10.6)
	[Min; Max]	[2.8;58.0]	[5.8;27.8]	[3.9;51.2]	[2.0;58.0]	[2.0;58.0]
Sex	Female (%)	73 (52.9%)	44 (44.0%)	66 (52.8%)	101 (54.6%)	284 (51.8%)
	Male (%)	65 (47.1%)	56 (56.0%)	59 (47.2%)	84 (45.4%)	264 (48.2%)

**Table 2:** Last parasiticide treatment received by dogs, according to dog owners.

Type of treatment	France	Greece	Italy	Spain	Total
Repellent	45 (32.8%)	30 (30.0%)	58 (46.4%)	93 (49.0%)	226 (40.9%)
Non-repellent	72 (52.6%)	0 (0%)	62 (49.6%)	36 (18.9%)	170 (30.8%)
None	20 (14.6%)	70 (70.0%)	5 (4.0%)	61 (32.11%)	156 (28.3%)

**Table 3:** Rapid test results according to country.

Results	France	Greece	Italy	Spain	Total
Negative	134 (97.1%)	81 (81%)	111 (88.8%)	179 (96.8%)	505 (92.2%)
Positive	4 (2.9%)	19 (19%)	11 (8.8%)	6 (3.2%)	40 (7.3%)
Missing	0 (0%)	0 (0%)	3 (2.4%)	0 (0%)	3 (0.5%)

A univariable analysis was performed to investigate which factors were associated with the rapid serology test results. The prevalence observed in Greece was significantly higher than that in both France and Spain (*p* < 0.001), and the use of plasma was associated with a significantly lower risk of the outcome compared to serum (*p* = 0.010). Body weight, sex, and age showed no significant influence on the rapid test outcomes (Table 4). From this analysis, two factors were selected for the multivariable analysis: The material used to perform the test (plasma vs. serum) and country. In the multivariable model, only country remained a significant predictor after a backward selection approach.

**Table 4:** Univariable mixed-effects logistic regression model displaying the risk ratio and 95% confidence interval.

Parameter	Comparison	Risk ratio	95% CI	*P*-value
Sex	Female/Male	1.09(0.33)	[0.60;1.96]	0.780
Body weight	<20 kg/> = 20 kg	1.28(0.41)	[0.69;2.38]	0.430
Age	<= 5 years/>5 years	0.79(0.25)	[0.43;1.45]	0.450
Material use	Plasma/Serum	0.17(0.10)	[0.04;0.70]	0.010
Country	France/Greece	0.15(0.08)	[0.04;0.60]	<0.001
	France/Italy	0.32(0.18)	[0.07;1.39]	0.190
	France/Spain	0.89(0.57)	[0.17;4.57]	1.000
	Greece/Italy	2.11(0.75)	[0.85;5.23]	0.150
	Greece/Spain	5.86(2.65)	[1.84;18.69]	<0.001
	Italy/Spain	2.78(1.37)	[0.78;9.89]	0.160

## Discussion

CanL represents one of the most prominent vector-borne diseases of concern among veterinarians, pet owners, and public health authorities in endemic areas, mainly due to its impact on canine health and the dog’s role as a reservoir. Dog owners are not always aware of the need for regular screening for *Leishmania* spp. infection, nor of the importance of applying appropriate parasiticides with activity against sand flies. The interpretation of parasiticide use in relation to *Leishmania* test positivity was limited by the absence of key information on treatment compliance and the timing of the last administration. As the exact dates of treatment administration were not documented, it was impossible to draw conclusions about the potential contribution of the treatments. Collecting this information would have been highly valuable, as it would have enabled a reliable comparison between treatments administered during the sand fly season and infection status, thereby allowing more accurate conclusions regarding the protective effect of repellent products.

Veterinary professionals play an important role in educating citizens and pet owners about the severity of canine and human leishmaniasis and in advising appropriate diagnostic tests and preventive treatments. Clinical manifestations of CanL range from mild cutaneous lesions that appear at the site of sand fly parasite deposition to a general involvement of many organs characterized by lymph node enlargement, weight loss, and cutaneous, renal, articular, and ocular signs. Clinically affected dogs represent only a small proportion of the total infected canine population, while latent asymptomatic infections may persist for years, as demonstrated in many longitudinal studies (10–12). Outcomes of *Leishmania infantum* infection range from a subclinical condition to clinical stages of increasing severity (12, 13). Progression to overt disease is preceded by antibody seroconversion, which leads to an uncontrolled concentration of globulins that bind large amounts of *Leishmania* antigens, resulting in the formation of circulating immune complexes (CICs). Deposition of CICs in specific organs results in glomerulonephritis, vasculitis, uveitis, myositis, and polyarthritis. This is the reason why it is crucial to make an early diagnosis of infection to limit the progression of the disease and to reduce the dog’s role as a reservoir by applying appropriate therapeutic and preventive strategies. Accordingly, rapid tests for the detection of antibodies against *Leishmania* spp., which are widely used in routine practice across many countries of the European Mediterranean basin, should be considered the first screening step in the diagnosis approach to leishmaniasis. Dogs testing positive on rapid serological assays should undergo confirmation of their infection status using quantitative serological methods, such as the specific immunofluorescence antibody test (IFAT) or the enzyme-linked immunosorbent assay (ELISA), in combination with PCR-based methods. These steps are crucial because asymptomatic infected dogs are known to constitute an important animal reservoir for *Leishmania* parasites.

Infectiousness of dogs to competent phlebotomine vectors is influenced by several factors, with the severity of clinical signs being the most important (4). Sick dogs often present, in many cases, various skin lesions that have been associated with increased infectiousness, while the ability of sand flies to acquire parasites from the intact skin of AS infected dogs under natural conditions is not fully elucidated (14). In this context, it is crucial to identify AS dogs as early as possible. The assessment of AS infectiousness is lacking simple tests, and xenodiagnosis remains the only method for evaluating the potential transmissibility of the parasite. However, it requires the availability of a competent sand fly laboratory colony and the sedation of animals undergoing the procedure. Although xenodiagnosis remains the reference method for assessing the infectiousness of dogs, there are several alternative diagnostic tests available to veterinarians for detecting infection in clinically healthy dogs. Serological tests, such as the IFAT and ELISA, are considered the gold standard for CanL diagnosis (15–17). However, accurate staging of the disease often requires a combination of serological and molecular tests (13, 18). Despite this, owners’ compliance with the execution of diagnostic protocols can be an issue, particularly in AS dogs. In these cases, the use of rapid tests as an in-clinic screening tool could be pivotal for suspecting infection in healthy dogs presented to the clinic for periodic health check-ups or routine vaccination programs. As demonstrated in this study, a significant number of AS dogs living in endemic areas tested positive on the rapid test, with a median value of 7.3%. The dogs examined across the different study sites were homogeneous in terms of age and sex, except for BW. However, data analyses showed that BW did not influence the rapid test results.

All dogs included in the study were privately owned and presented in good general condition to clinics for annual health examinations. This non-random sampling design was chosen for two reasons: (i) to select AS dogs living in *Leishmania-*endemic areas and (ii) to avoid overlap between the seroprevalence results of privately owned AS dogs and those of stray dogs housed in private or public kennels. Although the latter category of dogs is of considerable epidemiological interest, it is not comparable to owned dogs, as stray dogs are exposed to a higher risk of infection due to factors such as an outdoor lifestyle and suboptimal medical management.

Incidentally, the comparison of serological results according to the type of sample used for the rapid test revealed significant differences. More positive dogs were identified when using serum compared to plasma, despite both matrices being equally recommended by the manufacturer. Tests performed on serum accounted for more than twice the number of tests performed on plasma (362 versus 169) and were predominantly used in regions with the highest number of positive dogs in the study (Greece and Italy), which may partially explain the observed difference.

## Conclusion

The results of this study highlight once again the pivotal role of veterinarians in endemic areas for CanL in raising dog owners’ awareness about this vector-borne disease. Specifically, veterinarians play a key role in performing periodic screening tests in clinically healthy dogs, improving knowledge of CanL seroprevalence, highlighting the potentially epidemiological role of Leishmania-seropositive healthy dogs, and encouraging dog owners to use parasiticides active against sand fly bites to reduce the risk of *L. infantum* transmission.

## Data Availability

The raw data supporting the conclusions of this article will be made available by the authors, without undue reservation.
